# Enhanced Biological Response of AVS-Functionalized Ti-6Al-4V Alloy through Covalent Immobilization of Collagen

**DOI:** 10.1038/s41598-018-21685-3

**Published:** 2018-02-20

**Authors:** Parsa Rezvanian, Rafael Daza, Patricia A. López, Milagros Ramos, Daniel González-Nieto, Manuel Elices, Gustavo V. Guinea, José Pérez-Rigueiro

**Affiliations:** 1 Centro de Tecnología Biomédica. Universidad Politécnica de Madrid, 28223 Pozuelo de Alarcón (Madrid), Spain; 2 Departamento de Ciencia de Materiales, ETSI Caminos, Canales y Puertos. Universidad Politécnica de Madrid, 28040 Madrid, Spain; 3 Biomedical Research Networking Center in Bioengineering, Biomaterials and Nanomedicine (CIBER-BBN), Madrid, Spain; 4 Departamento de Tecnología Fotónica y Bioingeniería, ETSI Telecomunicaciones. Universidad Politécnica de Madrid, 28040 Madrid, Spain

## Abstract

This study presents the development of an efficient procedure for covalently immobilizing collagen molecules on AVS-functionalized Ti-6Al-4V samples, and the assessment of the survival and proliferation of cells cultured on these substrates. Activated Vapor Silanization (AVS) is a versatile functionalization technique that allows obtaining a high density of active amine groups on the surface. A procedure is presented to covalently bind collagen to the functional layer using EDC/NHS as cross-linker. The covalently bound collagen proteins are characterized by fluorescence microscopy and atomic force microscopy and their stability is tested. The effect of the cross-linker concentration on the process is assessed. The concentration of the cross-linker is optimized and a reliable cleaning protocol is developed for the removal of the excess of carbodiimide from the samples. The results demonstrate that the covalent immobilization of collagen type I on Ti-6Al-4V substrates, using the optimized protocol, increases the number of viable cells present on the material. Consequently, AVS in combination with the carbodiimide chemistry appears as a robust method for the immobilization of proteins and, for the first time, it is shown that it can be used to enhance the biological response to the material.

## Introduction

Titanium (Ti) and titanium alloys are among the most-commonly used biomaterials for hard tissue replacement^1,2^. This choice is due to their good corrosion resistance, good mechanical properties and excellent biocompatibility^1,3,4^. However, the difficulty for establishing a direct contact with adjacent tissues has been a drawback for their application as implants^5^. Hence, a variety of surface engineering techniques have been proposed that, by modifying the morphological and chemical properties of the surface, intend to modulate the response of the body to the implanted titanium and to improve the interaction between the implant and bone tissue^2,6,7^. A promising approach consists of immobilizing biomolecules, so that specific responses in the tissue in contact with the implant are induced^8,9^. Extra cellular matrix (ECM) proteins are usual targets in this approach, since their biological function implies an intimate interaction with cells^10–12^.

Collagen is the most abundant protein component of the ECM and was proven to support proliferation and differentiation of different cell lineages^13,14^. Fibroblast and osteoblast adhesion onto collagen is believed to be regulated by the recognition of the protein through specific proteins, such as α_1_β_1_ and α_2_β_1_ integrins^15,16^. Consequently, collagen has been used as a coating in an attempt to improve the biological properties of metallic biomaterials^17–19^. Although adsorption is the most simple procedure to cover a surface with any given protein, adsorbed proteins are not strongly bonded to the surface and variations in the environment can cause them to be easily desorbed^20,21^. Moreover, adsorbed collagen is highly susceptible to biodegradation^16^, and adsorption may lead to conformational changes which can affect its properties^22^.

Covalent immobilization of proteins provides an alternative approach to modulate the biological response to materials. Covalent immobilization creates a stable link between the protein and the surface that is resistant to environmental changes and increases the stability of the immobilized protein^23^. However, there is a need to functionalize the surface of the metallic substrate with appropriate reactive groups. Subsequently, proteins can be covalently bound to these reactive groups using a cross-linker. Different strategies were proposed for the covalent immobilization of biomolecules on metallic surfaces which include different functionalization procedures and active proteins. For instance, bone morphogenic proteins (BMP) have been immobilized on titanium after functionalizing the material through liquid phase silanization using a solution of 3-aminopropyltriethoxysilane (APTS) in toluene and carbonyl diimidazole as cross-linker^24^. Other examples of functionalization protocols include the functionalization in liquid phase by APTS in toluene using glutaraldehyde as cross-linker to immobilize alkaline phosphatase and rat albumin on titanium^25^, functionalization with isocyanatopropyl-triethoxysilane (IPS) in toluene in combination with 1-ethyl-3-(3-dimethylaminopropyl)carbodiimide (EDC) for immobilization of collagen on stainless steel^16^ and functionalization with polydopamine using EDC as crosslinker for the immobilization of collagen on titanium^22^. The selection of the crosslinker was recognized as a critical point in the whole functionalization process since, for instance, it was found that glutaraldehyde might act as a cytotoxic agent *in-vitro*^26^. In contrast, carbodiimide cross-linkers are water-soluble and the excess molecules can be relatively easily removed^27–29^.

In this study, we use activated vapor silanization (AVS), a method developed by the authors that allows the surface functionalization of a wide range of materials^30–32^, in combination with the EDC/NHS chemistry to covalently immobilize collagen on Ti-6Al-4V samples. The covalently immobilized collagen is characterized and its stability on the surface demonstrated. Finally, by performing *in-vitro* cell cultures, we address the hypothesis that the covalently immobilized collagen is able to enhance the adhesion and proliferation of mesenchymal stem cells on functionalized Ti-6Al-4V samples compared with the bare material.

## Results

### Collagen adsorption

**Figure 1:**
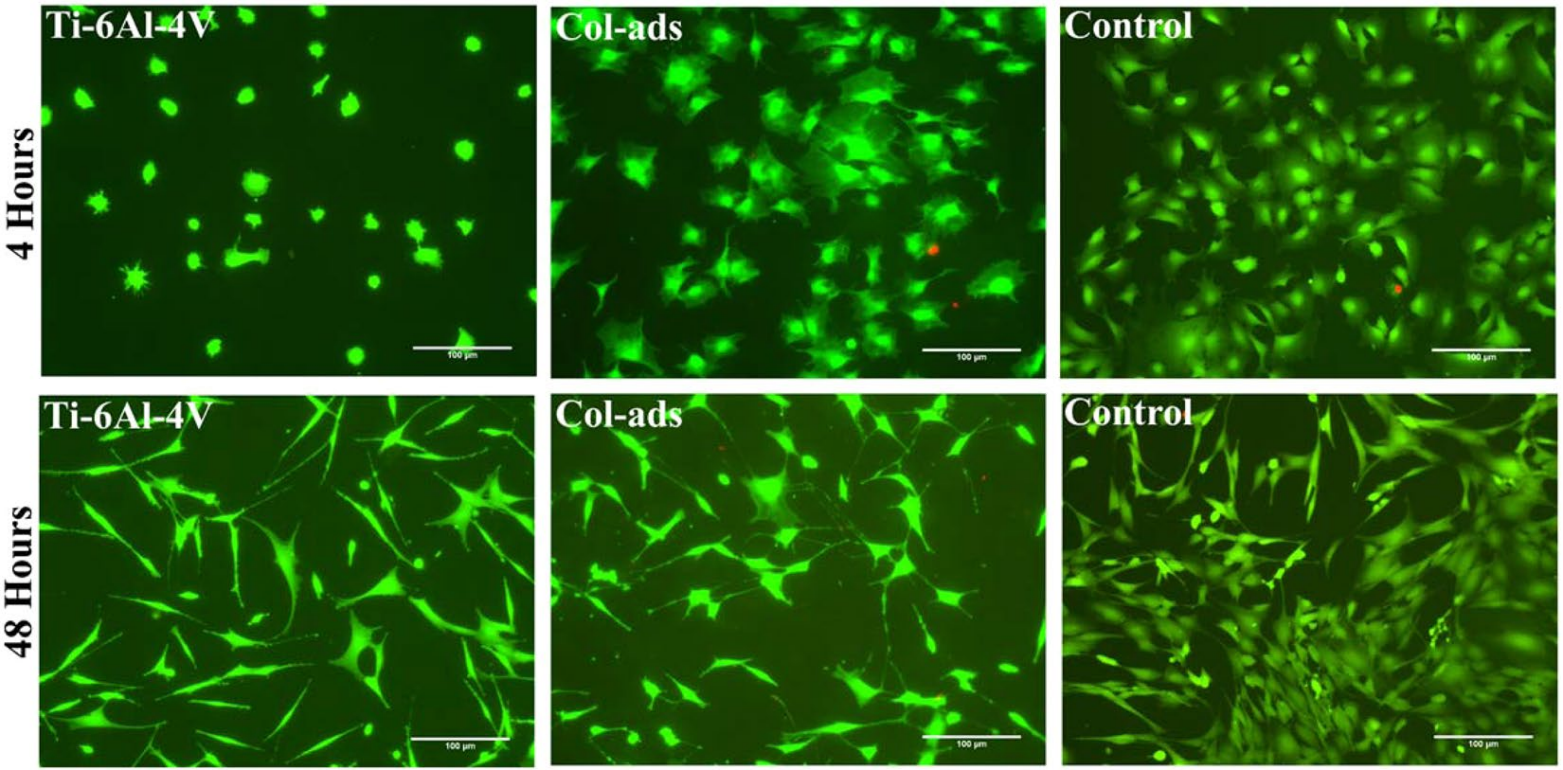
Morphology of the MSC cells cultured on bare Ti-6Al-4V, adsorbed collagen on Ti-6Al-4V (Col-ads) and plastic control samples after 4 and 48 hour of seeding. Viable cells are stained green while the dead cells appear as red dots. Scale Bar: 100 μm.

As a means to investigate the effect on cell behavior of a collagen type I coating on Ti-6Al-4V samples, collagen type I was adsorbed onto non-functionalized (bare) Ti-6Al-4V substrates, leading to the formation of a film (Col-ads). Figure 1 shows the calcein AM/propidium iodide stained MSC cells on bare Ti-6Al-4V, Col-ads and polystyrene control samples after 4 and 48 hours of seeding.

**Figure 2:**
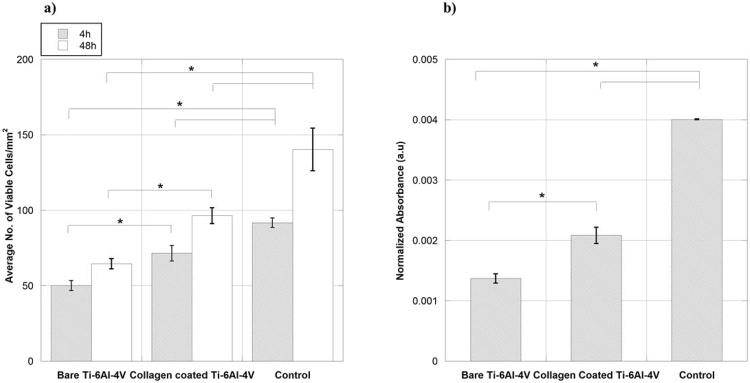
Number of cells obtained by (**a**) cell counting from micrographs after 4 and 48 hours of seeding, and (**b**) XTT reaction after 48 hours of seeding for MSC cells on bare Ti-6Al-4V, collagen adsorbed on Ti-6Al-4V (Col-ads) and control samples. *Denotes p < 0.05.

After 4 hours of seeding the cells are adhered to all samples, however the morphology of cells on Col-ads and control samples show a higher degree of extension of cellular processes, probably related with a dynamic cytoskeletal rearrangement, compared to the cells adhered on Ti-6Al-4V samples. The number of adhered cells on Col-ads samples is higher than that on bare Ti-6Al-4V, but lesser than on the controls (Fig. 2a). This trend in the number of cells on each sample remains consistent after 48 hours of seeding, demonstrating improved cell adhesion after 4 hours of seeding and a higher number of cells after 48 hours on Col-ads samples compared to bare Ti-6Al-4V. XTT assay performed at 48 hours after seeding also confirms the results of cell counting (Fig. 2b).

### Covalent immobilization of collagen

**Figure 3:**
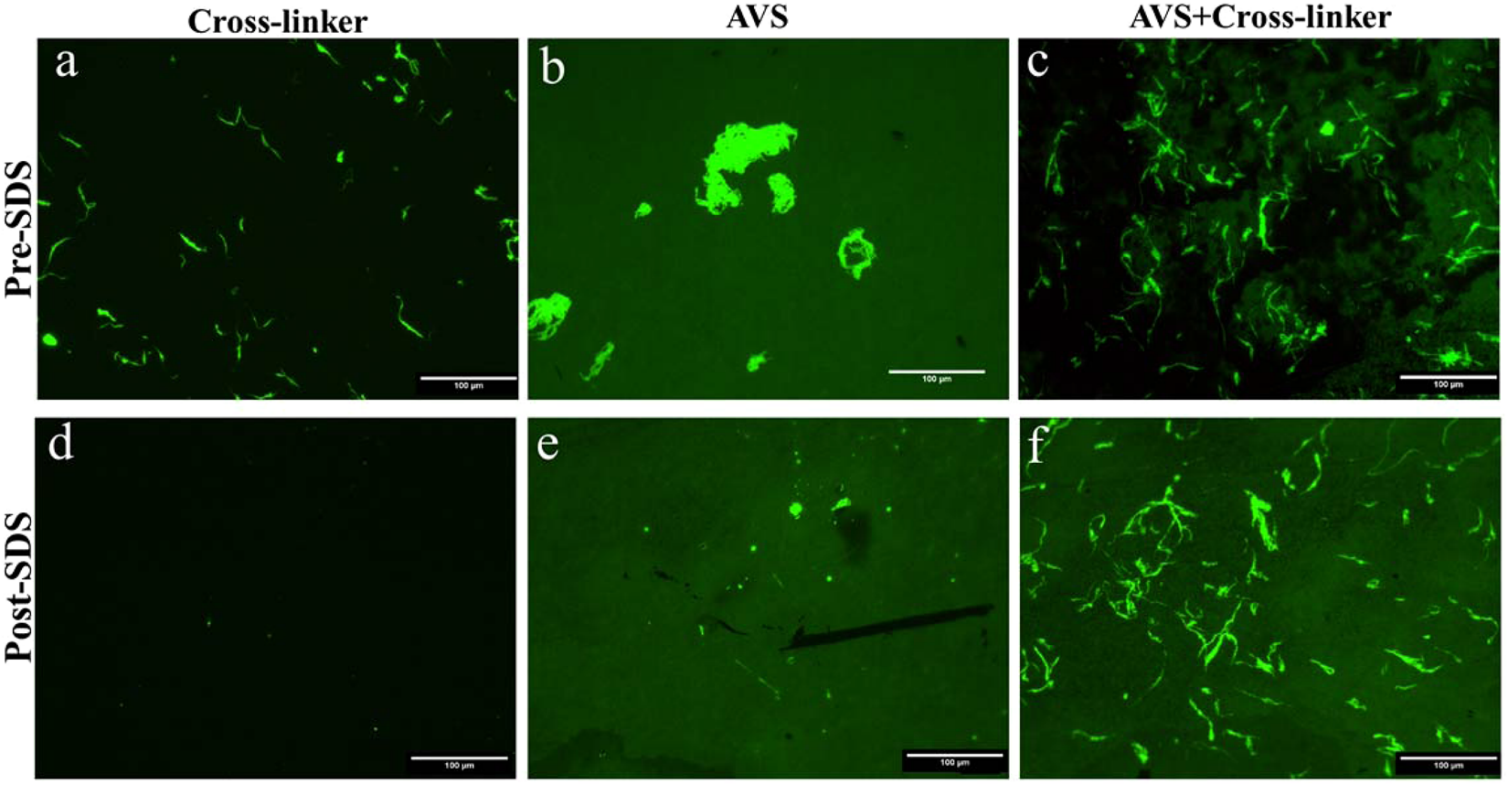
Fluorescence microscopy images of samples incubated with FITC-tagged collagen on (**a**) bare Ti-6Al-4V incubated with EDC/NHS cross-linker before SDS treatment and (**d**) after SDS treatment, (**b**) AVS-functionalized Ti-6Al-4V without EDC/NHS cross-linker before SDS treatment and (**e**) after SDS treatment, and c) AVS-functionalized Ti-6Al-4V incubated with EDC/NHS cross-linker (Col-imm-f) before SDS treatment and (**f**) after SDS treatment. Scale Bar 100 μm.

Figure 3 shows fluorescence microscopy images of a functionalized Ti-6Al-4V sample incubated with collagen and EDC/NHS (Fig. 3c) compared to a bare (non-functionalized) Ti-6Al-4V sample, which was incubated with the same FITC-tagged collagen and EDC/NHS solution (Fig. 3a), and a functionalized Ti-6Al-4V sample incubated with FITC-tagged collagen, but without the addition of EDC/NHS cross-linker in the solution (Fig. 3b). Collagen aggregates are apparent on the surface of all three samples, but the number of aggregates is higher on Col-imm-f (Fig. 3c) samples. After the treatment with SDS, it can be seen that fluorescence remains practically unchanged in the Col-imm-f (Fig. 3f), while a significant reduction is observed in the other two samples (Fig. 3d,e).

**Figure 4:**
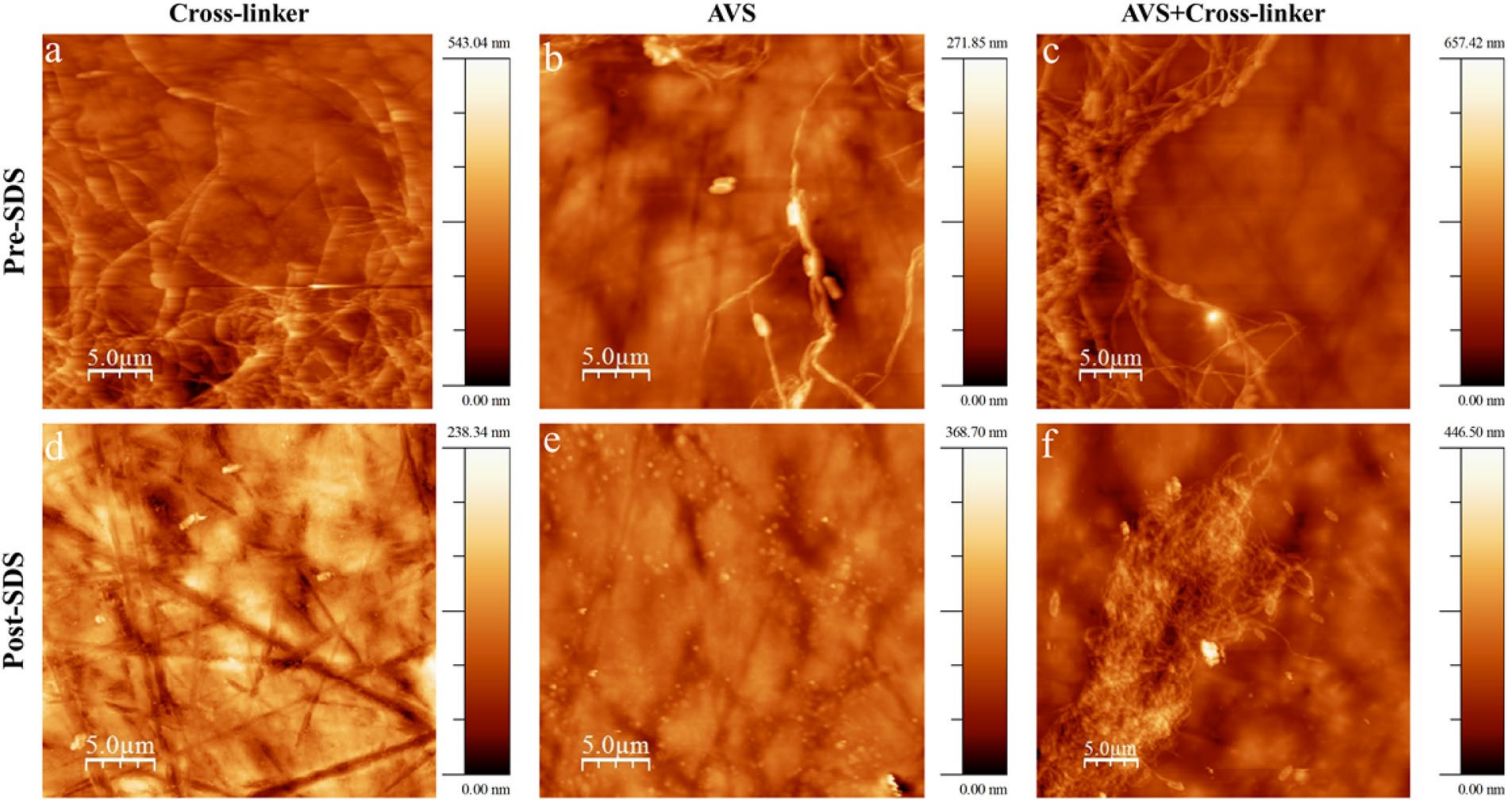
AFM topography images of samples incubated with FITC-tagged collagen on (**a**) bare Ti-6Al-4V incubated with EDC/NHS cross-linker before SDS treatment and (**d**) after SDS treatment, (**b**) AVS-functionalized Ti-6Al-4V without EDC/NHS cross-linker before SDS treatment and (**e**) after SDS treatment, and (**c**) AVS-functionalized Ti-6Al-4V incubated with EDC/NHS cross-linker (Col-imm-f) before SDS treatment and (**f**) after SDS treatment. Scale Bar 5 µm.

Figure 4 shows AFM topography images of the aforementioned samples. Before SDS treatment, the collagen aggregates are clearly visible in all three samples. After the SDS treatment, a significant removal of non-immobilized collagen is observed in micrographs d and e, but not in the functionalized Ti-6Al-4V sample incubated with collagen and EDC/NHS (f). AFM topography images of a bare and functionalized Ti-6Al-4V are also shown in the Supplementary data Fig. 1 for comparison.

The ability of SDS to remove adsorbed protein from the bare material was assessed by forming films of FITC-tagged collagen on Ti-6Al-4V samples and, subsequently, incubating them with 10% SDS solution in PBS. The fluorescence microscopy images can be seen in Supplementary data Fig. 2. It can be seen that after the incubation with SDS almost all the FITC-tagged collagen is removed from the substrate. On the other hand, the SDS treatment did not have any adverse effects on the amine functional layer. Since the layer is observed to be completely stable after the SDS treatment (Supplementary data Fig. 2).

**Figure 5:**
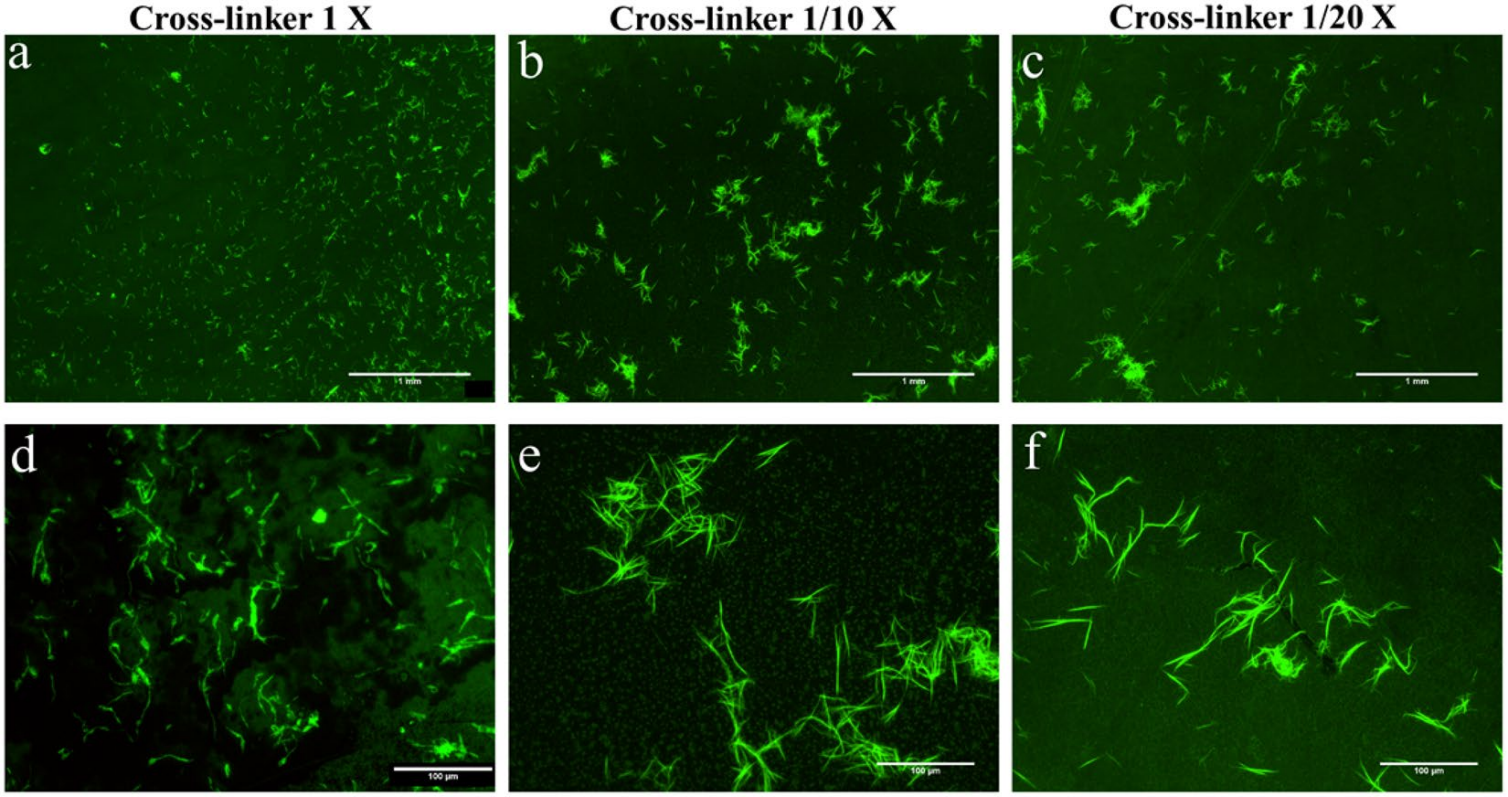
Fluorescence microscopy images of functionalized Ti-6Al-4V samples incubated with collagen and different concentrations of EDC/NHS. (**a**,**d**) 1X concentration of EDC/NHS (**b**,**e**) 1/10X concentration of EDC/NHS (**c**,**f**) 1/20X concentration of EDC/NHS. (**a**,**b**,**c**) Scale Bar 1 mm and (**d**,**e**,**f**) Scale Bar: 100 μm.

**Figure 6:**
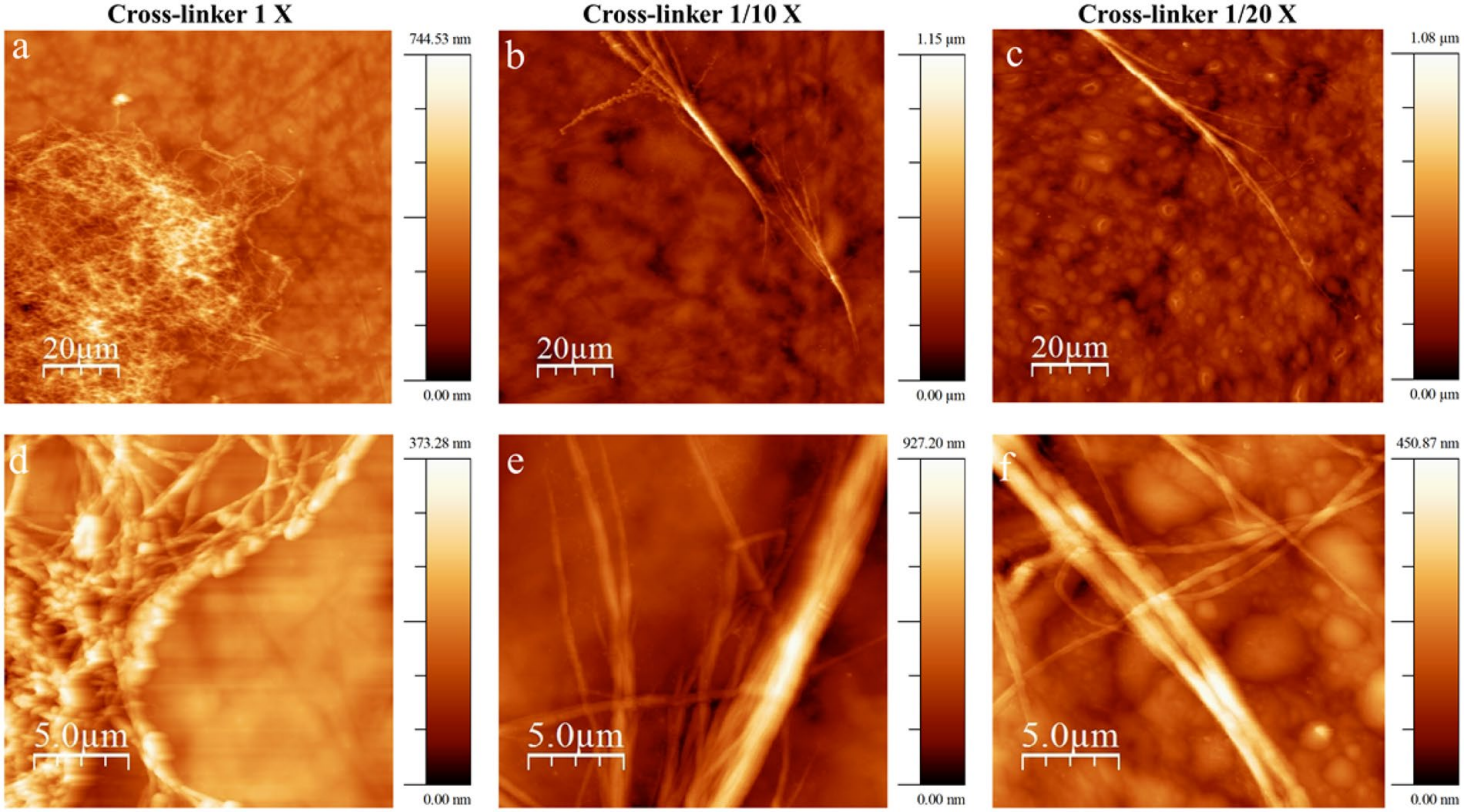
AFM topography images of functionalized Ti-6Al-4V samples incubated with collagen and different concentration of EDC/NHS at different scan sizes (**a**,**d**) 1X concentration of EDC/NHS, (**b**,**e**) 1/10X concentration of EDC/NHS, and (**c**,**f**) 1/20X concentration of EDC/NHS.

In order to determine the effect of EDC/NHS cross-linker concentration on the covalent binding of the protein, collagen was immobilized on functionalized Ti-6Al-4V samples, changing the concentration of the EDC/NHS cross-linker while keeping constant the concentration of collagen. Figure 5 shows fluorescence microscopy images of the FITC-tagged collagen covalently immobilized on functionalized Ti-6Al-4V samples using EDC/NHS concentrations of 2.5/0.63 mg/ml (1X), 0.25/0.063 mg/ml (1/10X) and 0.125/0.0315 mg/ml (1/20X). Although, the fluorescence content appears higher on the 1X sample, even in case of the lowest EDC/NHS concentration (1/20X), a significant fluorescence is still observable on the surface of the samples. The presence of immobilized collagen even in the 1/20X samples is confirmed by the AFM topography images as shown in Fig. 6. The amount of immobilized collagen on 1X, 1/10X and 1/20X samples was determined quantitatively by the Micro-BCA assay leading to the values: 0.20 ± 0.04 µg/mm^2^ (1X), 0.114 ± 0.009 µg/mm^2^ (1/10X), 0.117 ± 0.009 µg/mm^2^ (1/20X).

**Figure 7:**
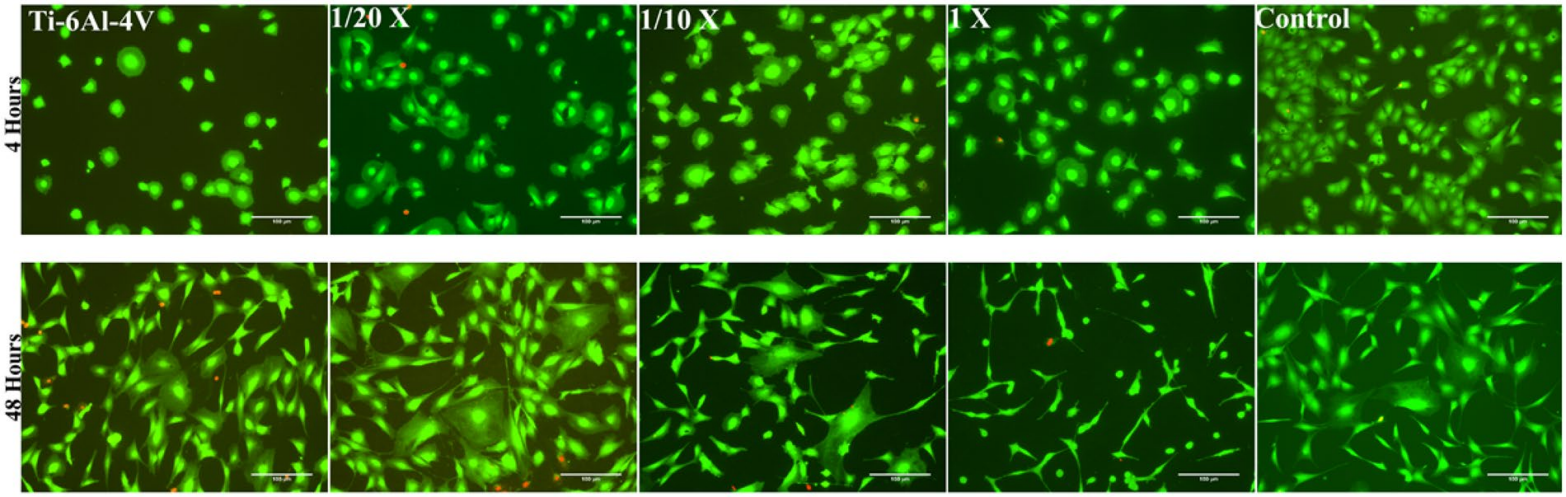
MSC cells adhered on bare Ti-6Al-4V, functionalized Ti-6Al-4V with immobilized collagen containing 1X, 1/10X and 1/20X concentrations of EDC/NHS, and control sample at 4 and 48 hours after seeding. Viable cells are stained green, while the dead cells appear as red dots. Scale Bar 100 μm.

**Figure 8:**
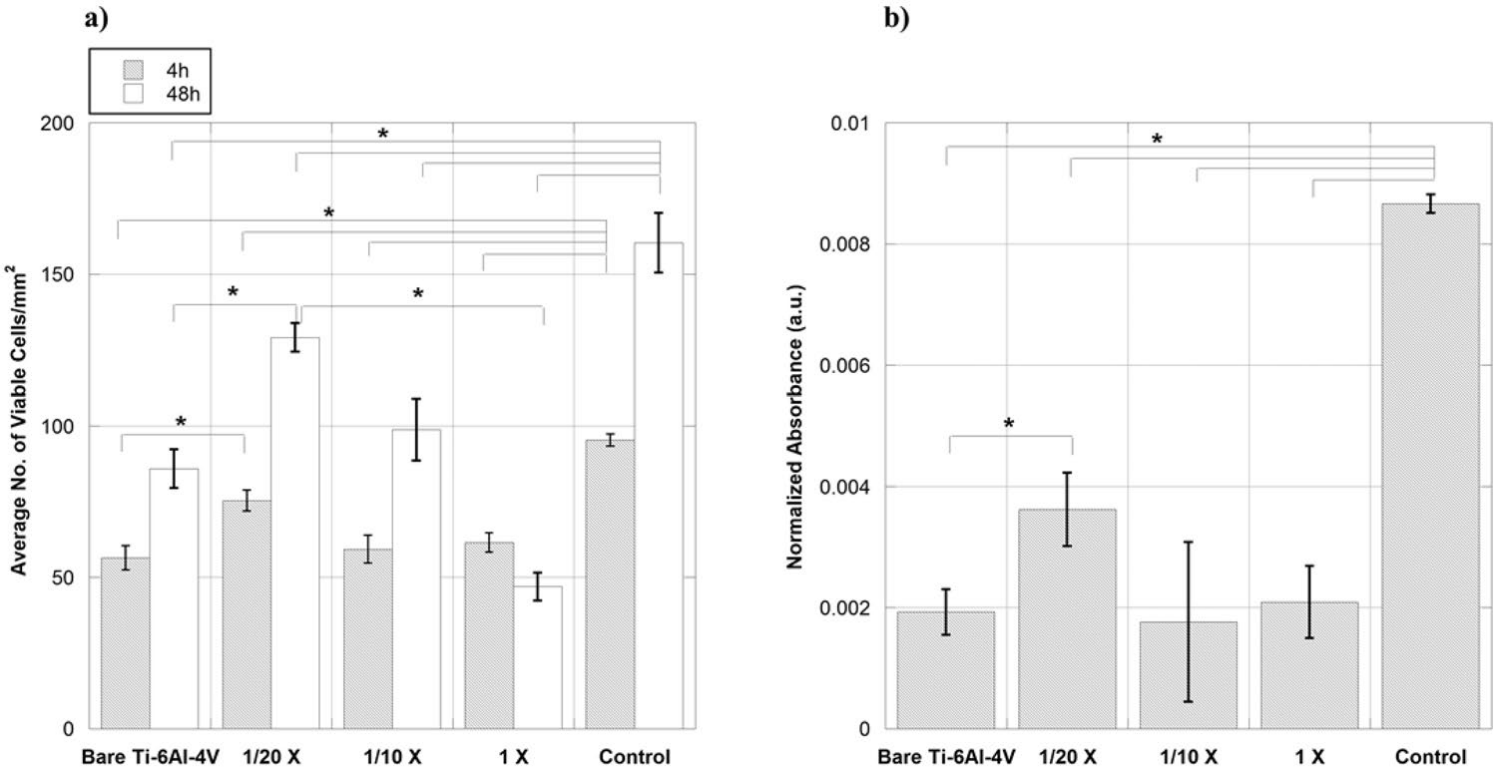
Number of MSC cells obtained by (**a**) cell counting from micrographs after 4 and 48 hours of seeding and (**b**) XTT reaction at 48 hours after seeding on bare Ti-6Al-4V, functionalized Ti-6Al-4V with immobilized collagen containing 1X, 1/10X and 1/20X concentrations of EDC/NHS, and control sample. *Denotes p < 0.05.

In order to assess the cytocompatibility and the effect of covalently immobilized collagen on the surface of Ti-6Al-4V samples on cell behavior, *in-vitro* cell cultures were performed. The morphology of MSC cells on 1X, 1/10X and 1/20X compared to bare Ti-6Al-4V and polystyrene controls at 4 and 48 hours after seeding can be seen in Fig. 7. The number of cells counted on each sample (Fig. 8a) demonstrated that at either time period, 1/20X sample had significantly more attached cells compared to bare Ti-6Al-4V. The better adhesion of 1/20X group was translated into a higher number of viable cells at later time points, as confirmed by XTT assay (Fig. 8b).

**Figure 9:**
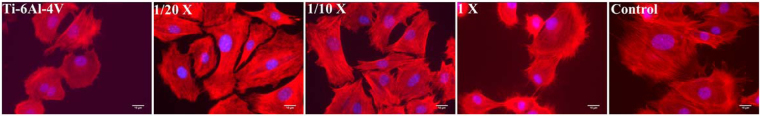
Fluorescence microscopy images of Phalloidin/Hoechst stained MSC cells adhered on bare Ti-6Al-4V, functionalized Ti-6Al-4V with immobilized collagen containing 1X, 1/10X and 1/20X concentrations of EDC/NHS, and control sample after 4 hours of seeding. Scale Bar 20 μm.

**Figure 10:**
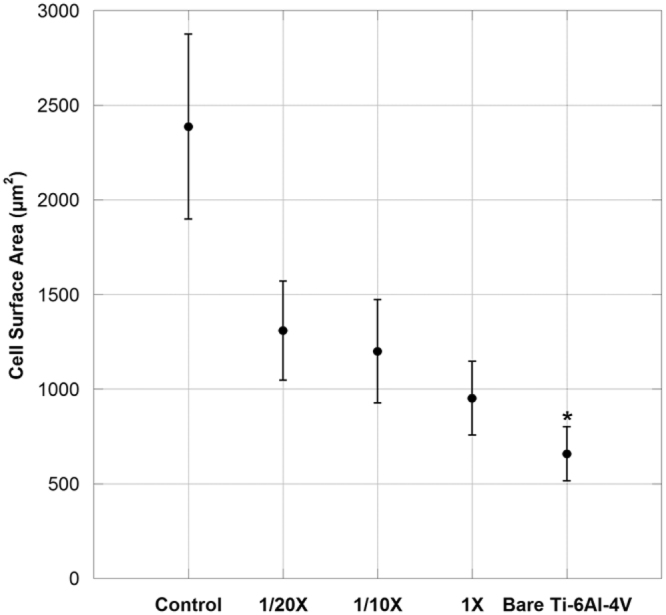
Surface area of MSCs on bare Ti-6Al-4V, functionalized Ti-6Al-4V with immobilized collagen containing 1X, 1/10X and 1/20X concentrations of EDC/NHS, and control sample after 4 hours of seeding. *Denotes p < 0.05 with respect to the control sample.

Although at 4 hours after seeding the cells were adhered on all the samples, those on the samples containing immobilized collagen showed a more widespread morphology compared with bare Ti-6Al-4V samples. This observation was further supported by phalloidin/hoechst staining (Fig. 9) and cell surface area measurements (Fig. 10).

Although the phalloidin/hoechst analysis was not extended to longer incubation times, it is apparent from Fig. 7 that cells on 1X samples show a spherical morphology at 48 hours after seeding, in contrast to all the other samples which mostly exhibit cells with polygonal shapes.

## Discussion

Although in a previous paper we showed the capability of AVS to effectively functionalize Ti-6Al-4V substrates, the enhancement of the biological response of biomaterials requires two additional steps: (1) using an adequate crosslinking chemistry to immobilize the biomolecule on the material, and (2) making a choice with regard to the biomolecule (or biomolecules) to be immobilized.

Collagen type I is a convenient choice due to its ubiquitous presence in the ECM and availability. Consequently, there are several studies on cell adhesion and proliferation on collagen coated Ti-alloy substrates. Ao *et al*. reported improved cell adhesion of human MSCs on collagen type I coated titanium after 4 hours of culture time^33^. Roehlecke *et al*. also demonstrated the enhanced cell adhesion and proliferation for collagen type I adsorbed onto titanium compared to bare titanium^34^, suggesting a possible positive effect of collagen on cell cycle evolution. It was also found that a collagen coating on titanium alloys promotes long and short term cell adhesion and proliferation of rat calvarial osteoblasts^20^. However, some contradictory results can be also found in the literature. Bierbaum *et al*. observed no improvement on cell proliferation after 2 days of culturing rat calvarial osteoblast on collagen type I coated Ti-6Al-4 V^35^.

Therefore, we decided to perform an initial assessment of the influence of a collagen coating on the survival and proliferation of murine MSC cells using collagen type I simply adsorbed on Ti-6Al-4V samples (Col-ads samples). As it can be seen in Fig. 2, the number of viable cells present on Col-ads samples is significantly higher than on bare Ti-6Al-4V at both time periods.

The enhanced cell adhesion and higher final number of cells on Ti-6Al-4V with an adsorbed collagen layer led to the development of a protocol to covalently immobilize the protein on the functionalized samples. Among the different possible choices, the EDC/NHS chemistry was employed due to its simplicity. Covalent immobilization proceeds by the initial formation of a semi-stable reactive ester between the –COOH groups of the glutamate or aspartate residues present in collagen^36,37^, and the NHS molecule. When the semi-stable ester comes into contact with an amine located on the functionalized surface of Ti-6Al-4V, a stable amide bond is created between the protein and the amine group^37–40^.

The stability of the covalently immobilized collagen was examined by exposing the samples to an SDS solution. It was observed that, in contrast to the results that showed the removal of adsorbed collagen on bare titanium samples upon SDS washing, no significant effect was observed on the covalently immobilized collagen samples (Col-imm-f) (Fig. 3c and f). Additionally, it was checked that the functional layer was not altered by exposure to an SDS solution and keeps its integrity even after the treatment, as shown in Supplementary data Fig. 2.

The *in-vitro* biological response of samples with immobilized collagen was assessed by cell culture of MSCs (Fig. 7). As it can be seen, at 4 hours after seeding all the functionalized samples show a relatively high number of adhered cells. However, depending on the concentration of EDC/NHS used, the response of the cells after 48 hours of seeding was quite different. In 1X samples, the number of cells declined at 48 hours after seeding, suggesting an inhibited cell proliferation and lower cell survival. Besides, the morphology of the cells on 1X samples show a dominantly circular morphology. This anomalous morphology can be explained by a possible cytotoxic effect of the EDC/NHS cross-linker. Despite the fact that there are several available studies which report no cytotoxic effect of carbodiimide cross-linker^41–43^, EDC in high concentrations might be toxic. This effect could be the result of DNA crosslinking caused by EDC, which is membrane-permeable, and would lead to the disturbance of the cell cycle^44^. Concentrations between 10 mM (1.9 mg/ml)^37^ and 1 mg/ml^16^ were reported to produce cytotoxic effects.

In contrast to the 1X samples, the number of cells on 1/10X and 1/20X samples grew after 48 hours of culture. In particular, it was shown that 1/20X samples not only showed better cell adhesion after 4 hours of cell seeding (p = 0.0143), but also a higher number of attached cells at 48 hours after seeding compared to bare Ti-6Al-4V (p = 0.0002).

The assessment of biological response of the functionalized samples was completed with the study of cell morphology at short incubation times. It was observed that after 4 hours of seeding, the cells growing on all functionalized samples showed a slightly more extended cytoskeleton compared to bare Ti-6Al-4V. In particular, the measurements of the surface area of the cells adhered to the samples (Figs 9 and 10) demonstrated that the cells adhered on the plastic control samples possess the highest surface area, while those adhered on bare Ti-6Al-4V had a significantly lower surface area. The surface area of cells adhered on samples with immobilized collagen ranked between the polystyrene control and bare Ti-6Al-4V samples. It was observed that among these samples, the cells adhered on the 1/20X samples seem to possess a slightly higher average surface area.

The observed increase in the cell surface area of the functionalized samples compared with bare Ti-6Al-4V can be interpreted as the result of a more extended cell cytoskeleton which, in turn, can contribute to an improved cell attachment to the substrates. It has been reported that the initial extent of adhesion and spreading of the cell can influence its subsequent survival and proliferation^45^, so that this result is consistent with the higher number of viable cells found on 1/20X at longer incubation times.

In our case we have found an optimal concentration of EDC of 0.125 mg/ml (1/20X), when combined with the cleaning process indicated in the Materials and Methods section. The comparison of the tests on 1/20X, 1/10X and 1X samples highlights the importance of cleaning as one of the critical steps in the whole functionalization process as suggested previously^37,46^.

## Conclusion

In this work, we demonstrate the enhancement in cell adhesion and survival of MSCs on Ti-6Al-4V alloy induced by the covalent immobilization of collagen Type I on AVS-functionalized samples. An efficient protocol for the covalent binding of collagen to the amine functional layer based on the EDC/NHS chemistry is developed and complemented by a suitable cleaning process. The immobilization of collagen was characterized by fluorescence microscopy and atomic force microscopy, including its stability even after relatively harsh treatments, such as incubation with SDS.

The biological response was assessed by *in-vitro* cell culture experiments using MSCs. The results demonstrated that even relatively low concentrations of EDC/NHS could cause cytotoxicity for cells 48 hours after seeding. Finally, an optimized protocol based on a reduced EDC/NHS concentration and an exhaustive cleaning procedure was defined and proven to lead to an increase in the cell number on the material compared with those observed on bare Ti-6Al-4V samples.

Although these results illustrate the robustness of the whole process that combines AVS-functionalization and protein covalent immobilization, they are far from exhausting all the possibilities of the technique. In this regard, it can be stressed that the AVS process is compatible not only with a wide range of biomaterials and processing methods, but also with a variety of crosslinkers and biomolecules. We believe that this versatility offers a great opportunity to improve the biological response of numerous biomaterials significantly with a minimum modification of the fabrication procedures. This improvement, in turn, should reflect in an enhanced biocompatibility of implants and devices.

## Materials and Methods

### Substrate preparation

Ti-6Al-4V substrates with dimensions of 10 × 10 × 1 mm were cut from an ingot of commercially available alloy. Subsequently, the substrates were subjected to a polishing process using sandpapers grit No. 80, 400, 1200 and 4000 sequentially. Ultimately, the samples were cleaned by sonication in acetone, isopropanol and distilled water.

### Adsorption of collagen on bare Ti-6Al-4V substrates

In order to determine the effect exerted by the presence of collagen on Ti-6Al-4V substrates on cell adhesion/proliferation, initial tests with collagen adsorbed on bare (non-functionalized) Ti-6Al-4V substrates were conducted. Samples were incubated with a suspension of 2.5 mg/ml collagen (Rat tail Collagen type I, Corning) in PBS (Na_2_HPO_4_ 10 mM, KH_2_PO_4_ 1.8 mM, NaCl 137 mM, KCl 2.7 mM, pH = 7.4). A solution of NaOH (Panreac) 1 M was added dropwise to the collagen suspension to adjust the pH in the range of 6.0–8.0. Subsequently, 100 µl of the suspension was deposited on the surface of each bare Ti-6Al-4V substrate and the substrates were incubated at 37 °C for 2 h in a wet chamber followed by 1 h incubation at 37 °C in a dry chamber. Finally, the samples were immersed in distilled water for 1 h to rehydrate the collagen films. These samples were named Col-ads.

### Preparation of covalently immobilized collagen on functionalized Ti-6Al-4V substrates

#### Functionalization

Functionalization of Ti-6Al-4V substrates was performed using an Activated Vapor Silanization (AVS) equipment as described elsewhere^32^. Briefly, 3-Aminopropyltriethoxysilane (APTS, Fluka) is put inside a closed chamber and evaporated. The vapor is carried by an argon flux (BIP, Purity ≥99, 9997%) to an activation chamber where the temperature rises to 750 °C. Afterwards, the activated APTS vapor impinges on the substrates in the deposition chamber. Finally, the vapor phase is evacuated from the system by a rotary pump. After functionalization with AVS, the substrates were cleaned by sonication with acetone and isopropanol, rinsed with distilled water, dried using an argon flux and stored in air.

In the AVS process there are four controllable parameters: Evaporation temperature of the APTS (T_evap_), activation temperature of the APTS (T_act_), pressure of argon (P_Ar_) and time of deposition (t). For the functionalization of the substrates used in this study, the processing parameters were chosen as: T_evap_ = 150 °C, T_act_ = 750 °C, P_Ar_ = 2mbar, t = 20 min. This choice of parameters was based on a previous study^32^ that established the optimized functionalization conditions. This process results in a homogenous and dense amino-functionalized surface on Ti-6Al-4V substrates.

#### Labeling collagen with FITC

In order to assess the immobilization of the protein on the substrates, collagen molecules were tagged with fluorescein 5(6)-isothiocyanate (FITC, Fluka) which binds to the amine groups of the proteins. A suspension of collagen 2 mg/ml in PBS was prepared and FITC was added until a final FITC concentration of 0.5 mg/ml was reached.

Dialysis was employed in order to remove any free FITC molecules that had not reacted with the collagen molecules. The collagen-FITC solution was put inside a dialysis membrane (Snakeskin dialysis tubing, Thermo Scientific) and dialysis was performed against 1 L of PBS as medium. The medium was changed every 8 hours (three times in total). Before each change of the dialysis medium, 1 ml of the PBS medium was measured with an spectrophotomer (Halo RB-10) at a wavelength of 490 nm, and absorbance was compared to an absorbance-concentration standard curve to determine the concentration of free FITC molecules. The dialysis process was stopped when the concentration of FITC in the medium was determined to be below the resolution of the spectrophotometer (less than 0.005).

#### Covalent immobilization of FITC-labeled collagen

After the completion of the dialysis process, a solution of 4-Morpholine-ethanesulfonic acid (MES, Sigma-Aldrich) 0.2 M, pH = 6.0 was added to the collagen-FITC solution to a final concentration of collagen of 1 mg/ml. The functionalized samples were incubated with 1 ml of the solution of collagen in MES for 1 h. Subsequently, a solution of N-(3-Dimethylaminopropyl)-N′-ethylcarbodiimide hydrochloride (EDC, Sigma-Aldrich) and N-Hydroxysuccinimide (NHS, Aldrich) in MES 0.1 M, pH = 6.0 was added to the samples to obtain a final concentration of 2.5 mg/ml EDC, 0.63 mg/ml NHS (hereafter referred to as 1X concentration) and the incubation was continued for 4 h. Finally, the samples were removed from the solution and gently rinsed with distilled water to remove any non-adhered collagen. These samples were named Col-imm-f.

In order to determine the effect of the EDC/NHS cross-linker concentration on the process, different concentrations of EDC/NHS were used taking the 1X concentration as reference, and named 1/10X (one tenth of the reference concentration) and 1/20X (one twentieth of the reference concentration).

To eliminate the non-covalently bound collagen present on the surface, some samples were immersed in a solution of 10% sodium dodecyl sulfate (SDS, Fisher scientific) in PBS. The samples were sonicated initially for 1 h in the SDS solution, then the SDS solution was changed and the samples were incubated overnight.

#### Covalent immobilization of collagen on functionalized Ti-6Al-4V substrates for cell cultures (Col-imm)

Functionalized Ti-6Al-4 V samples were incubated with 75 µl of a 3.26 mg/ml collagen suspension in MES buffer (0.1 M, pH = 0.6) for 1 h. Subsequently, 25 µl of the EDC/NHS solution were added onto the substrates and the incubation continued for 4 h. The final concentrations were: Collagen 2.5 mg/ml, MES 0.1 M, (pH = 0.6), EDC 2.5 mg/ml and NHS 0.63 mg/ml. These concentrations correspond to the 1X concentration of EDC/NHS. In some samples, the initial concentration of EDC/NHS was modified correspondingly to produce the 1/10X and 1/20X samples.

Finally, in order to remove any free cross-linker molecules still present, the samples were cleaned in PBS (10 mM, pH = 7.4), MES (0.1 M, pH = 6.0) and Dulbecco’s Modified Eagle Medium (DMEM, pH = 7.4) for 5 h, 72 h and 24 h, respectively.

### Characterization

#### Fluorescence microscopy

In order to visualize the FITC-tagged collagen aggregates on the substrates, fluorescence microscopy was used. Samples were observed using an inverted microscope (Leica DMIRB) equipped with a digital camera (Leica DC100) at an emission wavelength of 520 nm for FITC. The observation conditions were: exposure time: 1.3 ms, gain: 2.1X and gamma: 0.68.

#### Atomic force microscopy

The topography of samples was studied by atomic force microscopy (Cervantes AFM, Nanotec S.L.). Profile data and roughness were analyzed using WSxM 5.0 software. AFM measurements were performed in air using a pyramidal cantilever (Olympus OMCL RC800, semi-angle 39°, nominal resonance frequency 69 KHz) in dynamic mode.

#### *In-vitro* cell cultures

Bone marrow (BM) murine mesenchymal stem cells (MSC) were used as cell model. Isolation and expansion of BM cells were performed on fibronectin-coated wells (Corning Inc., NY) in Iscove’s Modified Dulbecco’s Medium (IMDM, HyClone) supplemented with 20% of MSC stimulatory supplements (Stem Cell Technologies), 100 μmol/L 2-mercaptoethanol (Sigma), 100 IU/mL penicillin (Sigma), 0.1 mg/mL streptomycin (GIBCO), 2 mmol/L L- glutamine (GIBCO), 10 ng/mL human PDGF-BB (Peprotech), and 10 ng/mL rm-EGF (Peprotech). Adherent cell clusters were grown for a minimum of 5 passages. After this step, cells were regularly maintained in DMEM (HyClone) supplemented with 10% fetal bovine serum (FBS, HyClone) and 1% penicillin/streptomycin (Sigma). Cells were detached from the cultures dishes when necessary using Trypsin-EDTA (HyClone). All experiments were performed on MSC in passages 5 through 15. Prior to culturing, substrates were placed in p24 multiwells and were sterilized using UV irradiation for 20 min on each side. Cells were seeded on the substrates in a concentration of 50000 cells/ml (0.5 ml per well). The cells were maintained in an incubator at 37 °C in a humidified atmosphere of 5% CO_2_. Cells seeded on empty wells were used as controls and each experiment was done twice with duplicate samples.

Although one of the advantages of using MSC is their ability to differentiate to a number of cell lineages, including osteoblasts and chondrocytes^47^, it was considered that a proper analysis of this process was more adequate for a future experimental campaign. Following this rationale, the present study is focused on the survival and proliferation of MSC on biofunctionalized and bare Ti-6Al-4V substrates.

#### Cell Viability

To perform a live/dead assay, calcein AM /propidium iodide staining was used. Staining was done either at 4 or 48 hours after seeding. Cells were incubated with a combined solution of 1 μl/ml of calcein acetoxymethyl (Calcein AM, Life Technologies, 0.5 µg/µl in DMSO) and 1 μl/ml of propidium iodide (Sigma-Aldrich, 750 µM in PBS) in DMEM for 30 min. Cells were observed using a fluorescence microscope (Leica DMIRB). At least three representative images were captured of each sample.

#### Phalloidin/Hoechst staining

To observe the morphology of cells adhered on different substrates, actin filaments of the cells were stained with phalloidin tetramethylrhodamine B isothiocyanate (Phalloidin-TRITC, Sigma) and the nuclei counterstained with hoechst 33258 (Molecular Probes). Prior to staining, the samples were washed with PBS and cells were fixed in a solution of paraformaldehyde 4%. Afterwards, the fixed cells were rinsed with PBS once more, permeabilized using a solution of 0.1% triton X-100 and stained with a combined solution of 2 µg/ml phalloidin and 0.2 mg/ml hoechst in PBS. Finally, the samples were observed in a fluorescence microscope (Leica DMIRB) equipped with a digital camera (Leica DC100) at emission wavelengths 570 and 461 nm for phalloidin and hoechst, respectively.

In order to compare the level of cell spreading on each sample, the phalloidin/hoechst images obtained after 4 hours of seeding were analyzed. For each sample, 10 individual and perfectly separate cells were selected at random and the surface area of each cell was measured.

#### Cell proliferation assay

Cell proliferation on each sample after 48 hours of seeding was assessed by 2,3-bis-(2-methoxy-4-nitro-5-sulfophenyl)-2H-tetrazolium-5-carboxanilide (XTT, Applichem) following manufacturer’s instructions. The absorbance of each well was measured spectrophotometrically at 450 nm by using an ELX808 microplate reader (BioTeK). For comparative reasons, the absorbance values were normalized according to the surface area of each sample.

The maximum duration of the cell viability and proliferation assays was fixed to 48 h for two main reasons. Firstly, the results obtained at 48 h of incubation are less likely to be affected by the possible contact inhibition effect, when the cell density is high^48^. This effect might be important at longer incubation times when comparing the number of cells grown on the polystyrene control samples and those on the other substrates. Secondly, the large number of trials performed in order to obtain reliable data from the cell culture experiments, especially during the development of the cleaning protocol, converted the duration of these experiments in a critical parameter for the development of an efficient experimental campaign. A duration of 48 h for cell culturing was selected as a convenient time that allows studying the survival and proliferation of cells, while preventing contact inhibition and, simultaneously, keeping the time required for the experimental work within a reasonable limit. This range of cell culture times was used by other authors that assessed the *in vitro* response of cells to different materials^11,49,50^.

#### Protein quantification

The amount of proteins immobilized on 1X, 1/10X and 1/20X samples after completing the cleaning process was measured using Micro-BCA protein assay kit (Thermo Scientific). The samples were incubated with the working solution of the kit (A:B:C:PBS = 25:24:1:50) for 1 h at 60 °C followed by equilibration at room temperature for 30 min. The absorbance values were obtained at 562 nm and compared with a standard curve obtained from serial dilutions of bovine serum albumin in PBS. The assay was done with duplicate samples for each concentration.

### Image analysis

Image J software was used for image analysis, counting the number of viable cells and measuring cell surface area.

### Statistical analysis

To perform statistical analysis IBM SPSS Statistics 20 software was used. Statistical significance was determined using a one-way ANOVA followed by a Games-Howell post-hoc test. p < 0.05 was considered significant. All data are presented as mean value ± standard error.

### Data availability statement

No datasets were generated or analysed during the current study.

## Electronic supplementary material

Enhanced Biological Response of AVS-Functionalized Ti-6Al-4V Alloy through Covalent Immobilization of Collagen
