# Supplementary material for: Enhanced Biological Response of AVS-Functionalized Ti-6Al-4V Alloy through Covalent Immobilization of Collagen
Source: Sci Rep. 2018 Feb 20;8:3337. doi: 10.1038/s41598-018-21685-3 (PMC5820288; doi:10.1038/s41598-018-21685-3)
Supplement: Supplementary file 1 — Enhanced Biological Response of AVS-Functionalized Ti-6Al-4V Alloy through Covalent Immobilization of Collagen [file 41598_2018_21685_MOESM1_ESM.pdf]

Enhanced Biological Response of AVS-Functionalized Ti-6Al-4V Alloy through Covalent Immobilization of Collagen

SUPPLEMENTARY DATA

Parsa Rezvanian[1,2], Rafael Daza[1,2], Patricia A. López[1,2], Milagros Ramos[1,3,4] , Daniel González-Nieto[1,3,4], Manuel Elices[1,2], Gustavo V. Guinea[1,2,3], José Pérez-Rigueiro[1,2,3*]

1. Centro de Tecnología Biomédica. Universidad Politécnica de Madrid. 28223 Pozuelo de Alarcón (Madrid). Spain

2. Departamento de Ciencia de Materiales. ETSI Caminos, Canales y Puertos. Universidad Politécnica de Madrid. 28040 Madrid. Spain

3. Biomedical Research Networking Center in Bioengineering, Biomaterials and Nanomedicine (CIBER-BBN), Madrid, Spain

4. Departamento de Tecnología Fotónica y Bioingeniería. ETSI Telecomunicaciones. Universidad Politécnica de Madrid. 28040 Madrid. Spain

*Corresponding Author

SUPPLEMENTARY DATA

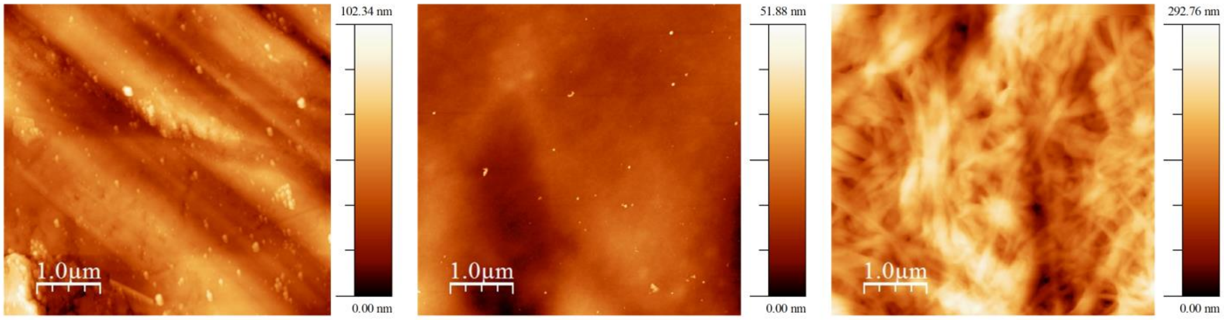

Supplementary Data Figure 1: AFM micrographs of a) non-functionalized Ti-6Al-4V sample, b) functionalized Ti-6Al-4V sample and c) functionalized Ti-6Al-4V sample with immobilized collagen.

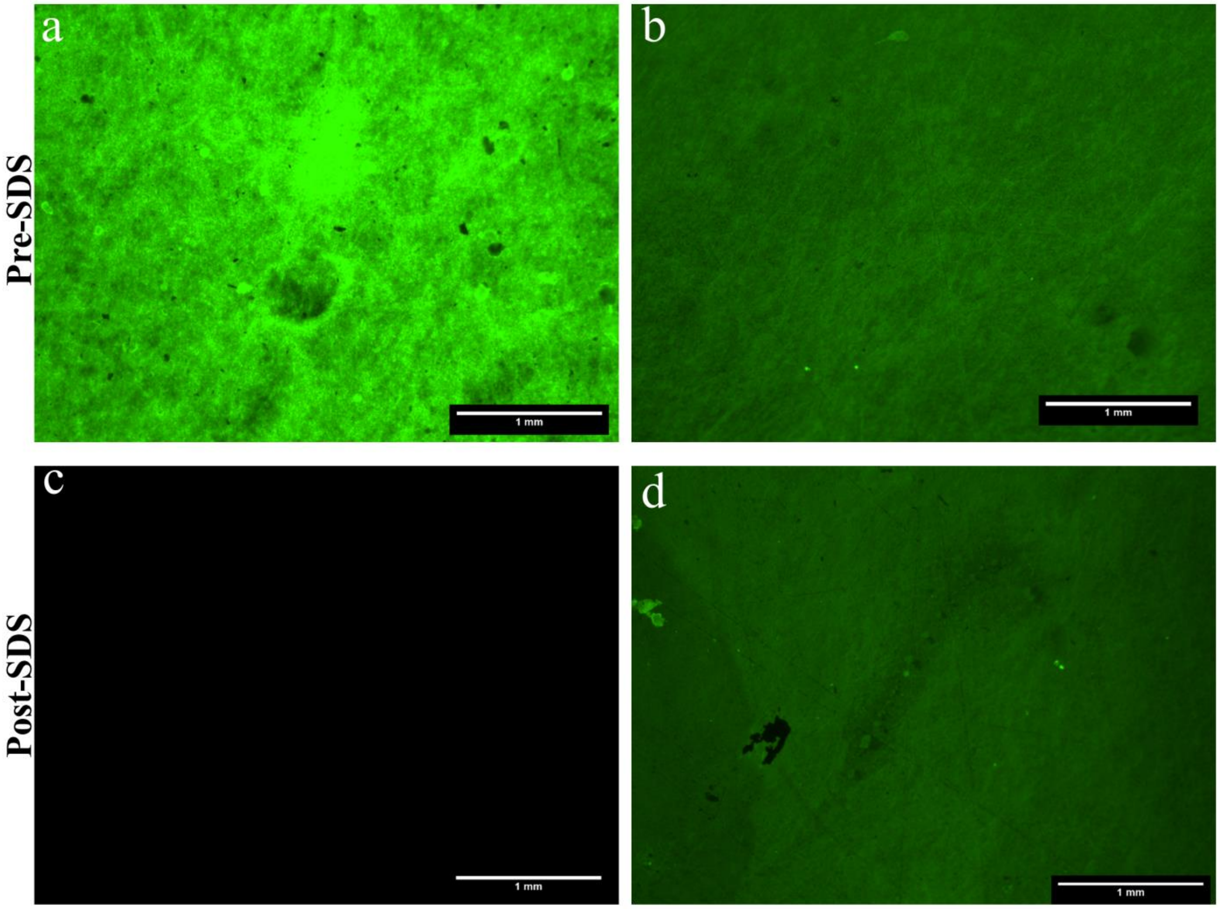

Supplementary Data Figure 2: Fluorescence microscopy images of FITC-tagged collagen film adsorbed on a) a non-functionalized (bare) Ti-6Al-4V substrate before SDS treatment and c) after SDS treatment compared with b) a functionalized Ti-6Al-4V samples dyed with FITC before SDS treatment and d) after SDS treatment. Scale Bar: 1mm.
